# Brief Intervention for Discontinuing Inappropriate Z-Hypnotic Use Among Older Patients in Primary Care: Protocol for a Cluster Randomized Controlled Trial With a Single Crossover

**DOI:** 10.2196/75670

**Published:** 2026-01-30

**Authors:** Tahreem Ghazal Siddiqui, Maria Torheim Bjelkarøy, Tone Breines Simonsen, Maria Lie Selle, Christofer Lundqvist

**Affiliations:** 1 Health Services Research Unit Akershus University Hospital Lørenskog Norway; 2 Department of Health Services Research Institute of Clinical Medicine, Campus Akershus University Hospital University of Oslo Lørenskog Norway

**Keywords:** Z-hypnotics, inappropriate prescription medication, older people, general practice

## Abstract

**Background:**

Older patients are frequent users of Z-hypnotics despite consensus recommendations against extended use. Inappropriate Z-hypnotic use among older patients is frequently reported, posing risks of side effects and dependence. Interventions have been mainly at the population level and through prescription regulations. There are few instruments helping general practitioners (GPs) deal with inappropriate use among individual patients.

**Objective:**

Through a randomized controlled trial (RCT), we aim to test the effectiveness of a behavioral brief intervention (BI) method used by trained GPs for reducing inappropriate Z-hypnotic use among their patients.

**Methods:**

We will conduct a double-blind RCT with a single crossover. Patients (aged >60 years) on participating GPs’ lists who are using Z-hypnotics inappropriately, do not have serious mental or physical disorders, and can provide valid informed consent are eligible. GPs randomized to the BI arm will be trained to administer the BI, and those randomized to business as usual (BAU) will not receive training. GPs’ patient lists will be screened for inappropriate Z-hypnotic users through an electronic questionnaire. The GP will be informed of patients who should be given an appointment and administered the BI. Untrained GPs will continue BAU. Randomization-blinded outcome evaluation will be conducted at 6 weeks, 6 months, and 1 year in both the study groups.

**Results:**

The main outcome is the proportion of patients with inappropriate Z-hypnotic use, comparing BI versus BAU, after 6 weeks. Secondary outcomes are cognitive function, pain, self-reported sleep evaluation, sleep efficiency (actigraphy) and quality of life, and change compared to baseline. We will also report on the characteristics of the screened GP patient population. Other variables are other medication use or polypharmacy, anxiety and depression, severity of dependence, and mortality.

**Conclusions:**

If RCT-level evidence demonstrates the effectiveness of the BI for reducing inappropriate Z-hypnotic use among older patients without worsening of secondary outcomes, this could be a simple, transferable intervention to implement on a larger scale among GPs, other physicians, and health workers.

**International Registered Report Identifier (IRRID):**

DERR1-10.2196/75670

## Introduction

### Background

The use of central nervous system–depressant medications with addictive potential such as opioids, benzodiazepines, and benzodiazepinelike hypnotics (Z-hypnotics) is widespread, and prescription-based use of these has become an increasing problem in many countries. Prescription-based use of such medications may pose an even greater risk among older patients, as is expressed in medication use consensus criteria and guidelines for older persons, such as the Beers Criteria for Potentially Inappropriate Medication Use in Older Adults and the Norwegian General Practice criteria [1,2]. Nevertheless, inappropriate use among older patients is highly prevalent, and despite guidelines, there has been no overall reduction in use [3]. The dominant medication group of those mentioned above among older patients in general practice living at home or in nursery homes is Z-hypnotics, used by 8% to 25% of patients [4-6]. Several studies suggest a clear increase in use over time, with older patients dominating the trend [7].

Although some studies have suggested successful methods for deprescription of Z-hypnotics among older people as well as patients in primary care, there is no consensus on effective deprescription methods [8,9]. This represents a knowledge gap in the area of individual intervention by general practitioners (GPs) against inappropriate Z-hypnotic use, and there is a need to demonstrate the effectiveness of an intervention to do this. Most previous studies have focused on both benzodiazepine and Z-hypnotic use and used different interventions, such as written information brochures and face-to-face interventions by GPs and pharmacists [10,11]. Intervention contents have ranged from abrupt termination of use to tapering over time with support regimens with different types of content [8,9]. Even though there are several similarities between the use of benzodiazepines and of Z-hypnotics among older patients, justifying similar intervention, there are also several differences that may justify the use of different approaches. First, Z-hypnotics in Norway are usually prescribed not on a fixed regimen but on a part pro re nata schedule, with the patients having access to prescriptions but being partly themselves responsible for when to actually take the medication and when not to. This means that, rather than starting with formal deprescription by the GP, the logical first step, also involving patient enablement in our setting, may be to focus on the medication-related behavior of the individual patient without formally removing access to the prescribed medication itself. In addition, it has been shown that, with regard to the use of Z-hypnotics among older patients, dose escalation is rarely observed. Although this does not exclude the possibility of induced tolerance, it has some advantages regarding termination of use in that tapering is usually not necessary from the standard low doses of Z-hypnotics used. This greatly simplifies the communication of the intervention as the aim can usually be to recommend complete termination of intake. However, the other side of the coin is that the risk of rebound insomnia and possible worsening of other aspects of Z-hypnotic drug use must of course be carefully monitored in a study aiming to achieve termination of use. In our previous studies, although they were conducted in a hospital-recruited population, we have demonstrated that cognitive function, pain, and quality of life are associated with use of central nervous system active medication, the dominating one of which in our population was Z-hypnotics, thus justifying that a study focusing on achieving a change in the use of Z-hypnotics should also assess these aspects.

### The Brief Intervention and Its Conceptual Framework

The original brief intervention (BI) based on initial screening followed by a short, structured intervention was developed for harmful alcohol use in the last decades of the 20th century. It was based on a validated screening instrument for harmful alcohol use and a structured set of communication skills adapted to be easily usable in any clinical setting, not least in primary care [12]. Similar interventions have since been adapted to many different settings and drugs and, most recently, to inappropriate use of prescribed or nonprescribed medication [9,13-16]. The original framework from the World Health Organization BI manual for alcohol and substance abuse [17] consisted of feedback responsibilities, advice, a menu of options, empathy, and self-efficacy [17-19], and in the preparatory work for this protocol, we adjusted this to our target population: older patients with Z-hypnotic use. The BI is communication based, with some elements from the motivational interviewing counseling method. The original BI manual provided some examples on parts of motivational interviewing in a consultation [17]. However, it does not fully cover the communication strategies and advice for framing information giving and exchanges between physicians and older patients. Thus, the BI was further adapted for the purpose of this protocol based largely on our preparatory work, and the method was piloted in a smaller study focusing on feasibility, logistics, and acceptability. We have previously adapted and successfully tested the BI scheme in interventions by GPs in the context of medication overuse headache (MOH) [20]. The BI for MOH proved to be an effective and acceptable tool for GPs and patients, thus enabling early intervention at the primary care level, where most patients with MOH are [20,21]. Our previous studies on the use of central nervous system active drugs among older patients admitted to hospitals from primary care showed that the use of such drugs is high in this population [22]. We have also shown their association with reduced cognitive function and quality of life, as well as with pain, addiction, and comorbidity [23-26]. The most common of these drugs in our hospital population in these studies were Z-hypnotics [22]. In Norway, health services are built based on the role of the GP as gatekeeper and primary health service contact for most home-living older patients. GPs are also responsible for prescribing the largest share of possibly addictive medication to these patients and, thus, are a key to interventions hoping to reach large groups of patients [27]. Even though GPs in Norway are trained to be restrictive regarding the prescription of such medications to older patients, not least based on the Norwegian General Practice (NORGEP) criteria, such use is increasing, with Z-hypnotics playing a dominant role [28]. On the basis of our previous experience, we have also adapted the BI scheme to users of Z-hypnotics, aiming to give structured advice enabling patients to reduce inappropriate use. The BI setup was piloted in a small case-series feasibility study, with feedback supporting a focus on the GP setting also in this case [29]. Our previous successful experience with the BI method in other settings with individual inappropriate medication use, as well as both our previous pilot study and our background studies on factors associated with inappropriate medication use, in particular Z-hypnotics, among older patients, suggests that the BI method is a suitable and promising candidate for an intervention study.

In this paper, we describe the protocol for a randomized controlled trial of the BI versus business as usual (BAU). The protocol was developed following the UK Medical Research Council guidelines for developing and evaluating complex interventions [30]. Our primary objective is to evaluate the effectiveness of a BI, conducted by trained GPs, for reducing inappropriate Z-hypnotic use in older patients. The main and secondary effects of the BI will be assessed at the patient level.

## Methods

### Design

We will conduct a double-blind, randomized, single-crossover controlled trial comparing the BI with BAU. The data will be collected at baseline and 6 weeks, 6 months, and 12 months after baseline.

### Trial Status

The first patient was recruited in October 2023. Patient recruitment has been completed as of May 2024. Data collection is ongoing until December 2024.

### Setting

**Figure 1:**
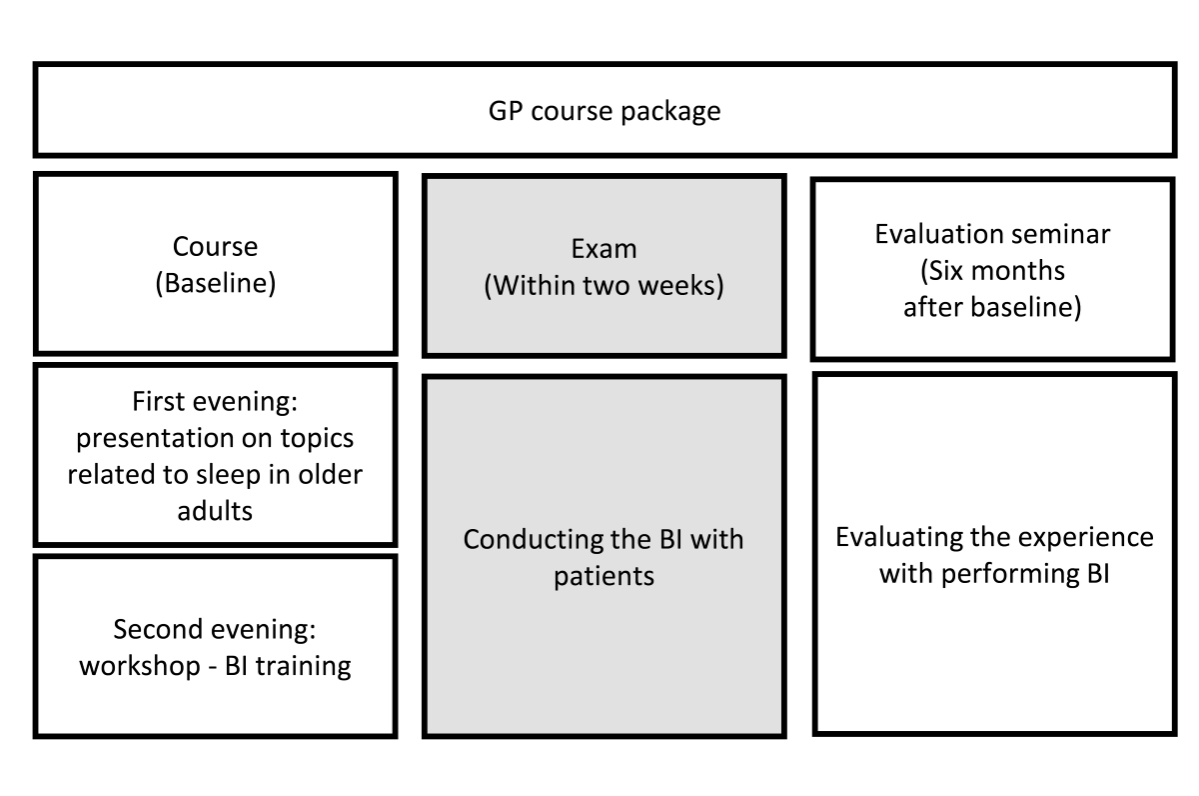
General practitioner course package and tasks. BI: brief intervention.

In the Norwegian health care system, the main point of entry for patients is via their GP, and it is intended that all patients in Norway be listed with a GP, who then has the primary responsibility for health care contacts, referrals to specialist health care, and prescriptions. The average GP list length is of approximately 1200 patients. GPs are, in most cases, self-employed, but the main funding comes from reimbursement from the national health insurance system, which is based on a fixed sum per patient on the list provided by the municipalities, a fee-for-service payment from the health insurance system, and a small copayment by the patient (approximately NOK 200 [approximately US$ 20]). Most GPs are specialized in general medicine, which requires recertification every 5 years. Recertification is based partly on continuous medical education courses, which are quality approved by the Norwegian Association for General Practice. This study will use such a course, offered for free to GPs and approved and certified by the board of the Norwegian Association of General Practice, to train GPs to administer the BI in their own practices and on their own patients (Figure 1).

### Recruitment, Randomization, and Blinding

GPs will be included by inviting them to a course on the treatment of sleep disorders among older patients. They will be informed that the course is also a part of a research study on an intervention regarding inappropriate use of sleep medications. An external statistician will then randomize the GPs to receive the course at 2 separate time points with an interval of 6 months between them. This will form the basis for the cluster randomization of the patients on the GPs’ lists; thus, patients will be randomized either to a GP participating in an earlier course or to a GP receiving the course later. Each GP and their patients will represent one cluster. We will avoid randomizing GPs belonging to the same GP center to different course groups to avoid cross-contamination of the intervention method. We will attempt to avoid selection bias, which would be expected through GPs selecting their own “suitable” patients, and will avoid collecting prescription patient data before patient consent. Thus, the study board will recruit the patients based on a short screening questionnaire, including written consent, to older patients (aged >60 years) on the patient lists of participating GPs. Self-reported sleeping problems, as well as use of sleeping medication, will be queried. After the course, GPs will receive a list of patients with self-reported Z-hypnotic use whom they should make an appointment with to administer the BI.

**Figure 2:**
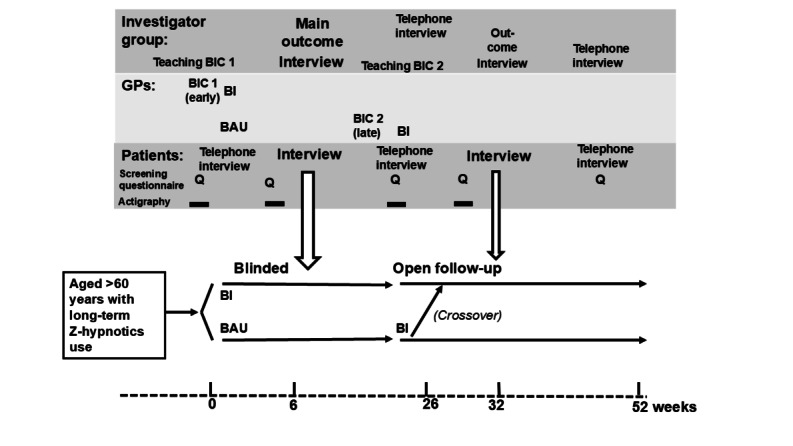
Outline of the study timeline. The upper half shows the tasks of the investigator team, general practitioners (GPs), and patients, and the lower part shows the timeline in weeks. Patients will be initially screened via a questionnaire and then receive self-report electronic questionnaires at specific time points (denoted as Q in the figure). GPs will be randomized to receive the behavioral intervention (BI) course (BIC) in 2 batches (BIC 1 will receive the course early, and BIC 2 will receive the course later) and administer the BI to their patients, who will be screened by the investigators at weeks 0 and 26, respectively (ie, BIC 1 patients at week 0 and BIC 2 patients at week 26). The late BI course will enable a crossover of initial business as usual (BAU) patients after 6 months. The main follow-up assessment (large arrows) will be performed by the investigators as an in-person appointment 6 weeks after the BI (or parallel BAU control).

We do not expect GPs randomized to the later course to change their handling of their Z-hypnotic users before their course participation, and thus, they will constitute the BAU arm during the first part of the study. Indeed, GPs randomized to the later course will not be informed of their included patients until they have received the BI training course to avoid a change in patient handling in advance, aiming to maintain a true BAU control group. Those randomized to the early course will constitute the active (BI) arm during this period. The second course will enable a single crossover of previous BAU patients who will then also receive the intervention, providing the possibility for enriched before-and-after BI intervention data (Figure 2).

### Patient Procedures

Data from patients will be collected as follows. First, GPs who consent to participate will deliver lists of names and contact addresses of all patients on their lists aged >60 years to the study board. The lists will contain no medical information and no personal ID numbers and will not be linked to patient data or hospital records. Second, patients on the list will be sent an email or SMS text message containing an information and consent document and a short screening questionnaire. Third, patients using Z-hypnotics will be recontacted in the same manner with an additional information and consent document inviting them to participate in a study focusing on sleep problems and sleep medication use patterns. Fourth, consenting patients will be contacted for a brief telephone interview before the GPs’ BI course (or in parallel to the course for BAU clusters) and administered an online questionnaire at baseline (Figure 2). Fifth, BI GPs will be informed of a maximal number of 3 patients whom they should make an appointment with to administer the BI. The BI will be administered as described below. Sixth, 6 weeks and 6 months after BI patients will receive the follow-up questionnaire, a new telephone interview will be performed, and patients will be invited to a brief face-to face interview either at the hospital or in the patients’ homes if they are unable to visit the hospital. Finally, additional telephone interviews and a follow-up online questionnaire will be conducted after 6 and 12 months. Incoming replies will be monitored (without addressing reply contents), and patient reminders will be given via telephone and SMS text messaging to reduce missing data and dropouts during follow-up. This will also enable patient safety monitoring.

All patient-derived data collection will be conducted via safe links through individual QR codes to safe Data Protection Impact Assessment–approved research servers.

### Intervention

The BI for inappropriate Z-hypnotic use taught to and administered by participating GPs consists of (1) an interview-based patient scoring system validated for detection of Z-hypnotic misuse and dependence in this population; (2) evidence-based and individualized information about Z-hypnotics and associated risks in our population, with special attention to the individual patient profile; (3) information about gains and difficulties with reducing inappropriate use (strong emphasis on information regarding expected rebound effects during the first couple of weeks after terminating use and the need to overcome this before improvement can be expected); (4) discussion of possible treatment goals for the patient; (5) formulation of a treatment plan, including goals to aim for, time frame, assumed difficulties and how to overcome them, and a plan for support from and follow-up by the GP office; and (6) summary and take-home message, including giving the patient a copy of the treatment plan.

Communication skills emphasized to GPs during the BI course will include being nonjudgmental and empathetic, using simple language and the teach-back method, formulating a written plan together with the patient, and emphasizing a supportive role.

### Inclusion and Exclusion

The inclusion criterion is self-reported use of Z-hypnotics >3 days per week for >4 weeks. Exclusion criteria are diagnosis of dementia, psychosis, major depression, or delirium; inability to provide informed consent; and insufficient Norwegian-language capacity to complete the tests.

### Data Collection and Follow-Up Time Points

Table 1 lists the main assessment time points of the study, as well as how data will be collected and which data we will collect for what purpose.

**Table 1:** Summary of main assessment time points and data to be collected.

Time point	Setting	Data to be collected	Purpose of the data
Before the intervention	Screening self-report online questionnaire to all patients on the GPs’^a^ lists Self-report online questionnaire Telephone interview	Self-report sleep problems and sleep medication use Basic sociodemographic data and data on anxiety and depression, pain, medication use, sleep, quality of life, and subjective cognitive function Telephone-administered cognition instruments	Basic information on GP population; screen for Z-hypnotic users for later intervention by the GP Baseline predictor variables Baseline cognitive function
Intervention	At the GP’s office	None	Outcome data
6 weeks after the intervention	Self-report digital questionnaire Face-to-face interview and cognitive tests	Anxiety and depression, pain, medication use, sleep, quality of life, and subjective cognitive function Cognitive function	Main outcomes: Z-hypnotic use, pain, and cognitive function
6 months after the intervention	Self-report digital questionnaire Face-to-face interview and cognitive tests Qualitative group interviews with GPs	Anxiety and depression, pain, medication use, sleep, quality of life, and subjective cognitive function Cognitive function Recorded group interviews	Main outcomes: Z-hypnotic use, pain, and cognitive function Qualitative data on acceptability of the use of the brief intervention by GPs
12 months after the intervention	Self-report questionnaire Telephone interview	Anxiety and depression, pain, medication use, sleep, quality of life, and subjective cognitive function Telephone-administered cognition instruments	Long-term outcomes, relapse, and dropout rate

^a^GP: general practitioner.

Follow-up time points will be chosen to enable catching possible rebound insomnia (6-week time point) and long-term relapse (up to 12 months). Missing data will be handled via last observation carried forward, providing at least one follow-up after the intervention.

### Actigraphy Subsample

A total of 20 patients will be issued with actigraphy (ActiGraph) wearables without screens, and we will assess their sleep patterns and daily activity patterns over 2 weeks before and after the BI or BAU. The wearables will be delivered to the patients, and the data will be uploaded to a local secure server at the next assessment time point.

### Qualitative Interviews

Six months after having tested the intervention with their assigned patients, GPs will be invited to a group interview to assess their experience using the BI method and the perceived acceptability among their patients. The interview will be based on an interview guide and will be video recorded and transcribed verbatim. The material will be qualitatively evaluated.

### Outcomes

#### Main Outcome

The main outcome, on which power has also been based, is the proportion of patients with inappropriate use of Z-hypnotics (defined as use ≥3 days per week for ≥4 weeks without consideration of dosage) in the BI arm compared to BAU.

#### Secondary Outcomes

Main and additional secondary outcomes are presented in Table 2 with the data collection methods and instruments.

**Table 2:** Secondary outcomes and the instruments we will use.

Outcome and measures		Instruments
**Main secondary outcomes**		
	Cognitive score	Montreal Cognitive Assessment (face-to-face and telephone versions) [31,32]
	Pain VAS^a^	100-mm VAS (numerical; 100 being the worst possible pain) [33]
	Sleep self-assessment	Global Sleep Assessment Questionnaire (11 items with response options of “never,” “sometimes,” “usually,” and “always”; item responses were converted to a common scale from 0 to 100, with a higher score indicating greater likelihood of presence of the disorder) [34]
**Additional secondary outcomes**		
	Sleep effectiveness	Actigraphy registration 2 weeks before and after the intervention (subgroup only)
	Quality of life	EQ-5D-5L (Norwegian population norm valuation); measured on a scale from 0 to 1, with 1 representing perfect health [35]
	Dependence-like behavior	Severity of Dependence Scale (5 questions, total score range from 0 to 15, with 15 being the most severe) [36]
	Additional cognitive assessments	Cognistat (numerical score from 0 to 84, with 84 being the best) [37] CFI^b^ (Norwegian self-assessed cognitive instrument) and FAS^c^ word score
	Polypharmacy	Number of drugs used regularly
	Anxiety and depression	Hospital Anxiety and Depression Scale (0-42, with 42 being the worst) [31,38]
	Willingness to change	Qualitative group interview
	Acceptability	Qualitative group interview

^a^VAS: visual analogue scale.

^b^Cognitive Function Instrument (Norwegian version).

^c^F-A-S word association test.

### Statistics

#### Power

On the basis of previous studies [39], and using a conservative estimate, we assume that the intervention may lead to a 30% detoxification rate in the intervention (BI) arm and that, at most, 2% of patients in the nonintervention (BAU) arm may detoxify over 6 months. With a power of 80% to detect a significant difference with α at .05, we would need to include 52 patients in all (26 per study arm). Power analyses for the 2 tentative main secondary outcomes with α adjusted to .025 (Bonferroni correction) suggest that, altogether, 384 patients are needed to detect cognitive improvement; this sample size is based on the difference between central nervous system–depressant drug users and nonusers in our previous studies [23]. Similarly, over 2000 patients would be needed to detect an improvement in pain [24]. We observed that we were unlikely to attain such recruitment figures and chose to focus on power related to the main outcome, which we suggested as being attainable. In addition, as the number of patients for each GP will be maximized to 3 (in most cases likely just 1-2 patients per GP), we assumed that the clustering effect would not significantly affect power.

#### Statistical Analyses

The main analyses will follow the intention-to-treat principle, but we will also perform per-protocol analyses based on which patients actually went through the BI intervention versus BAU. The primary analysis of the main outcome will assess difference in proportion of inappropriate Z-hypnotic users between the BI and BAU groups using the *z* test, which we also based our power calculations on. We will analyze secondary outcomes using linear and logistic regression for numerical and binary outcomes, respectively, as well as using relevant descriptive statistics.

### Ethical Considerations

All participation follows the Declaration of Helsinki, with written informed consent collected from both GPs and patients before any study-related procedures and collection of study data. Patients will receive the contact address of the study lead when signing the informed consent form and will be encouraged to make contact if problems or questions arise. In addition, GPs will have a running responsibility toward their own patients. Data will be stored on secure and Data Protection Impact Assessment–approved servers at the research institute. All procedures were assessed and approved by the Research Ethics Committee of South East Norway (REK 556653) and by the Data Inspectorate Office at Akershus University Hospital. Participants received no compensation.

The BI course was approved by the relevant board of the Norwegian Association of General Practice as providing part of the recertification points required for GPs’ continuous medical education.

### Dissemination of and Feedback on Results

In addition to publishing the results in relevant international peer-reviewed journals, we will ascertain deidentified feedback from the participating GPs and patients. Patients using actigraphy registration will also be able to receive their own registration assessment after completion of the study. Data will be anonymized and stored on safe servers as specified by the General Data Protection Regulation and approved by the research ethics committee and the data protection officer.

We are committed to publishing both positive and negative results We also intend to implement further dissemination of the BI course should the method yield positive results, as well as attempt to disseminate the results through basic teaching for GPs, other physicians, and medical students. Finally, it is a further goal to evaluate and implement parts of the course to give recommendations for treatment through relevant web platforms for health staff. User representatives and health care representatives in our steering committee will actively participate.

## Results

We expect to be able to show whether the BI is significantly better at reducing the number of older patients who use Z-hypnotics inappropriately than the nonintervention (ie, BAU), which is the main outcome of this study. In addition, we aim to demonstrate that there is no significant worsening of the main secondary outcomes of sleep (ie, no increase in reported insomnia), cognition, or pain as inappropriate Z-hypnotic use is reduced. Thus, these secondary outcome results may be viewed as assessing possible side effects of the intervention, which we suggest to be an important part of the overall assessment of gains versus losses of the intervention. Should side effects be increased after the intervention, especially if the primary outcome effect is not positive or is small, we will suggest that this is a strong argument against the intervention. However, if the main outcome effect is strong and we observe no downsides to this in terms of increased side effects, this will strengthen the argument for using the BI intervention in this patient population. As the recruitment process is vulnerable and has several steps, and we cannot completely eliminate a carryover effect (from trained GPs to untrained GPs, which would possibly reduce the effect size in the controlled part of the study), a single crossover for the control arm to receive the BI at a later stage was added to increase power and be able to more closely assess the timing of a possible change from the intervention based on when the intervention was delivered. We expect this crossover to also provide additional results on change in medication-related behavior and maintenance of such behavior over different periods up to 12 months. We expect the main results of the study to be published during winter 2025-2026.

## Discussion

As there is no established and generally accepted patient-centered intervention method to address inappropriate use of Z-hypnotics among older individuals, despite this being associated with several possible negative effects, there is a need for further evidence-based data on how to approach patients with possible inappropriate use. As this is a “hot topic” that is often debated, our aim is to provide randomized controlled trial–level evidence for the effect of a simple BI-based method that may, if proven effective, help GPs and other physicians address the issue with their patients.

We have chosen to base the intervention in primary care for several reasons. In Norway, GPs represent the most accessible part of the health care system, and almost all Norwegians are listed with a GP. GPs are charged with the responsibility of being gatekeepers for treatment and referral, when required, to the secondary, usually hospital-based, health services, as well as the follow-up of patients after hospital treatment. Therefore, the GP plays a key role in the prescription and termination of prescription, as well as in the assessment and follow-up of possible medication side effects for their patients. GPs are responsible for most prescriptions, whether these are first introduced by other specialists or by the GPs themselves. This is especially noticeable for prescribed drugs with a possible addictive potential [27]. For GPs who often have long-term care and responsibility for their patients, this may be a daunting task. Although simply the awareness of this responsibility and the central rules and recommendations regarding such medications have been suggested as effective means to limit inappropriate use in general, there is a paucity of structured instruments that may help GPs support individual patients in reducing inappropriate use. In addition, effective intervention at the primary health care level may be more cost-effective and follows the recommendations for treatment at the lowest effective treatment level, which is a central focus in the Norwegian decentralized health care system.

The GPs included in the study are from the southeastern health care region in Norway, which has both urban and rural GP settings and may be seen as reasonably representative. Participation of GPs will be via invitation to a course on sleep problems and sleep medication use to GPs in the entire region, although we cannot exclude that this may lead to some recruitment bias and recruit mainly GPs who are positive about a more restrictive use of Z-hypnotics. GPs need to obtain recertification points based on participation in several courses on different themes such as this one. Therefore, we expect to have a reasonably good mixture of representative GPs from our region as participants.

Regarding patient inclusion bias, we will attempt to reduce the risk of those most at risk of Z-hypnotic misuse dropping out and, thus, overrecruit patients who may be easy to “detoxify.” Information to patients stating that “detoxification” is a primary aim of a study certainly risks losing patients who are negative about such measures. Although research ethics requires that patients provide informed consent for participation, the way in which this information is formulated may be very important. In close correspondence with the responsible research ethics committee, we landed on a reasonable and ethical compromise regarding the formulation in this regard. We focused on individual evaluation and optimization of the medication to avoid side effects, as well as on individual follow-up of medication effects and side effects and re-evaluation if the effect of the intervention was perceived as negative.

The BI is a simple and short intervention that equips both patients and GPs with evidence-based knowledge and a systematic approach to discontinuing inappropriate Z-hypnotic use. If proven effective, a structured intervention that is simple to use and learn and easy to teach will likely be possible to implement at a larger scale, with the potential to be of benefit to many patients in primary care. Such implementation is a clear aim of our project, and we will use our network and user group to achieve this. We suggest that there is also a potential for implementation of similar individually focused interventions internationally, at least in countries with a similar primary health care system structure to the Norwegian one.

To avoid assessment effect and resulting change in behavior also in the control group, we have limited the amount of data collected in advance of the intervention, with no in-person visits in advance. We have chosen neutrally formulated questions and instruments assessing self-reported sleep, medication use, socioeconomic status, and pain in general. Specific questions focusing on the individuals’ use of Z-hypnotics and dependence-like characteristics only come after the intervention or control phase. However, this means that our preintervention data are limited.

Instruments used for data collection are based on our previous experience and have been validated for the age group and setting described and additionally validated by us in previous studies [40,41]. Self-reports on experienced sleep difficulties may be biased by patient expectations. Therefore, we have included a subsample of patients who will be monitored through actigraphy over a 2-week period before the intervention, as well as a similar period after the BI intervention or similarly timed for the BAU controls. Thus, we will be able to supplement self-reports with objective actigraphy measures of sleep efficiency and daytime activity.

Importantly, the design process of the intervention has been extensive and based on the recent guidelines from the UK Medical Research Council for the design of complex intervention studies [30], as well as on our own previous experience. In the process, a special emphasis was made on developing an intervention that was feasible to carry out by GPs in an ordinary GP consultation considering GP workload and available time. The feasibility study suggests that, with some minor logistics changes, we have succeeded in this [29]. However, this will be re-evaluated through qualitative interviews also as part of this study. We have also previously administered a similar BI in general practice for inappropriate medication use in the context of MOH with positive results, which also supports the viability of this study protocol [20,21,42].

## Abbreviations

BAU: business as usual
BI: brief intervention
GP: general practitioner
MOH: medication overuse headache
RCT: randomized controlled trial

## Data Availability

Though data protection policies preclude us from releasing identifiable data, fully anonymized data are available upon reasonable request to the authors and evaluation by the data inspectorate officer of the hospital.
